# Research progress of anaerobic ammonium oxidation (Anammox) process based on integrated fixed‐film activated sludge (IFAS)

**DOI:** 10.1111/1758-2229.13235

**Published:** 2024-03-05

**Authors:** Guang Li, Yunyong Yu, Xingyu Li, Hongsheng Jia, Xiaoning Ma, Prince Atta Opoku

**Affiliations:** ^1^ Key Laboratory of Songliao Aquatic Environment, Ministry of Education Jilin Jianzhu University Changchun China; ^2^ School of Environment Harbin Institute of Technology Harbin China

## Abstract

The integrated fixed‐film activated sludge (IFAS) process is considered one of the cutting‐edge solutions to the traditional wastewater treatment challenges, allowing suspended sludge and attached biofilm to grow in the same system. In addition, the coupling of IFAS with anaerobic ammonium oxidation (Anammox) can further improve the efficiency of biological denitrification. This paper summarises the research progress of IFAS coupled with the anammox process, including partial nitrification anammox, simultaneous partial nitrification anammox and denitrification, and partial denitrification anammox technologies, and describes the factors that limit the development of related processes. The effects of dissolved oxygen, influent carbon source, sludge retention time, temperature, microbial community, and nitrite‐oxidising bacteria inhibition methods on the anammox of IFAS are presented. At the same time, this paper gives an outlook on future research focus and engineering practice direction of the process.

## Graphical abstract

This study reviews the integrated fixed‐film activated sludge (IFAS) coupled anaerobic ammonium oxidation (Anammox) process including partial nitrification anammox, simultaneous partial nitrification anammox and denitrification, and partial denitrification anammox. And the factors affecting the IFAS‐Anammox process (dissolved oxygen (DO), influent carbon source, sludge retention time (SRT), temperature, microbial community, and nitrite‐oxidising bacterial (NOB) inhibition methods). Summarises the new findings of IFAS‐Anammox that offer possible help in solving the plague of Anammox.

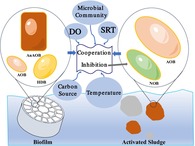

## INTRODUCTION

With the rapid development of human economic activities and urbanisation, more and more nutrients (nitrogen and phosphorus) from domestic and industrial wastewater are being discharged into receiving water bodies, causing serious problems such as eutrophication (Wang et al., 2019). Various treatment methods have been adopted to deal with these crises, among which the biological nitrogen removal (BNR) process is favoured for its simplicity and high efficiency (Zhou et al., 2018). Nevertheless, the conventional BNR process faces a huge consumption of energy and carbon sources, contrary to the goal of carbon neutrality. Autotrophic nitrogen removal (ANR) technology, which is low in energy consumption and does not require a carbon source, may resolve this contradiction (Leyva‐Diaz et al., 2020; Yang, Chen, et al., 2017).

The anaerobic ammonium oxidation (Anammox) process is a highly efficient and energy‐saving bioautotrophic nitrogen removal process, which is dominated by anaerobic ammonium‐oxidising bacteria (AnAOB). AnAOB oxidise ammonium (NH_4_^+^–N) directly to nitrogen (N_2_) using nitrite (NO_2_^−^–N) as an electron acceptor and without the need for aeration or organic carbon (Xie et al., 2022). Normally NH_4_^+^–N and NO_2_^−^–N are supplied by wastewater and ammonium‐oxidizing bacteria (AOB). However, AnAOB grows slowly (15–30 days ploidy time; Ma et al., 2016) and is sensitive to environmental fluctuations in dissolved oxygen (DO) and organic carbon concentrations, ammonium concentrations, and temperature, which leads to the fact that AnAOB is not easily retained and enriched in the system (Jetten et al., 1998). At the same time, AnAOB also competes with faster‐growing nitrite‐oxidising bacteria (NOB) for substrate and living space (Chen et al., 2023). These factors have become the biggest hindrance to the application of anaerobic ammonium oxidation technology in wastewater treatment plants. Suspended sludge, granular sludge, and biofilm can all be forms of biomass to be used to operate the Anammox process (Guo et al., 2020; Zhang, Zhang, Han, et al., 2020). Both granular sludge and biofilm can effectively retain and enrich functional bacteria, and both can form different gradients of DO and substrate from the outside to the inside, creating symbiotic conditions for aerobic AOB and anaerobic AnAOB Fields (Wu, Chen, et al., 2022). However, there are concerns about the stability of granular sludge (decomposition or uplift) in unfavourable environments (Wang, Li, et al., 2021). NOB colonising the surface of granular sludge and biofilms still compete with AOB and AnAOB for substrates (DO and NO_2_^−^–N) and living space in the system, which leads to a decrease in the nitrogen removal efficiency (NRE) of the system (Lackner et al., 2014; Zekker et al., 2012). Integrated fixed‐film activated sludge (IFAS) combines suspended activated sludge and attached biofilm in a system that has the advantages of both and is a superior choice for coupling Anammox (Mannina et al., 2017).

**FIGURE 1:**
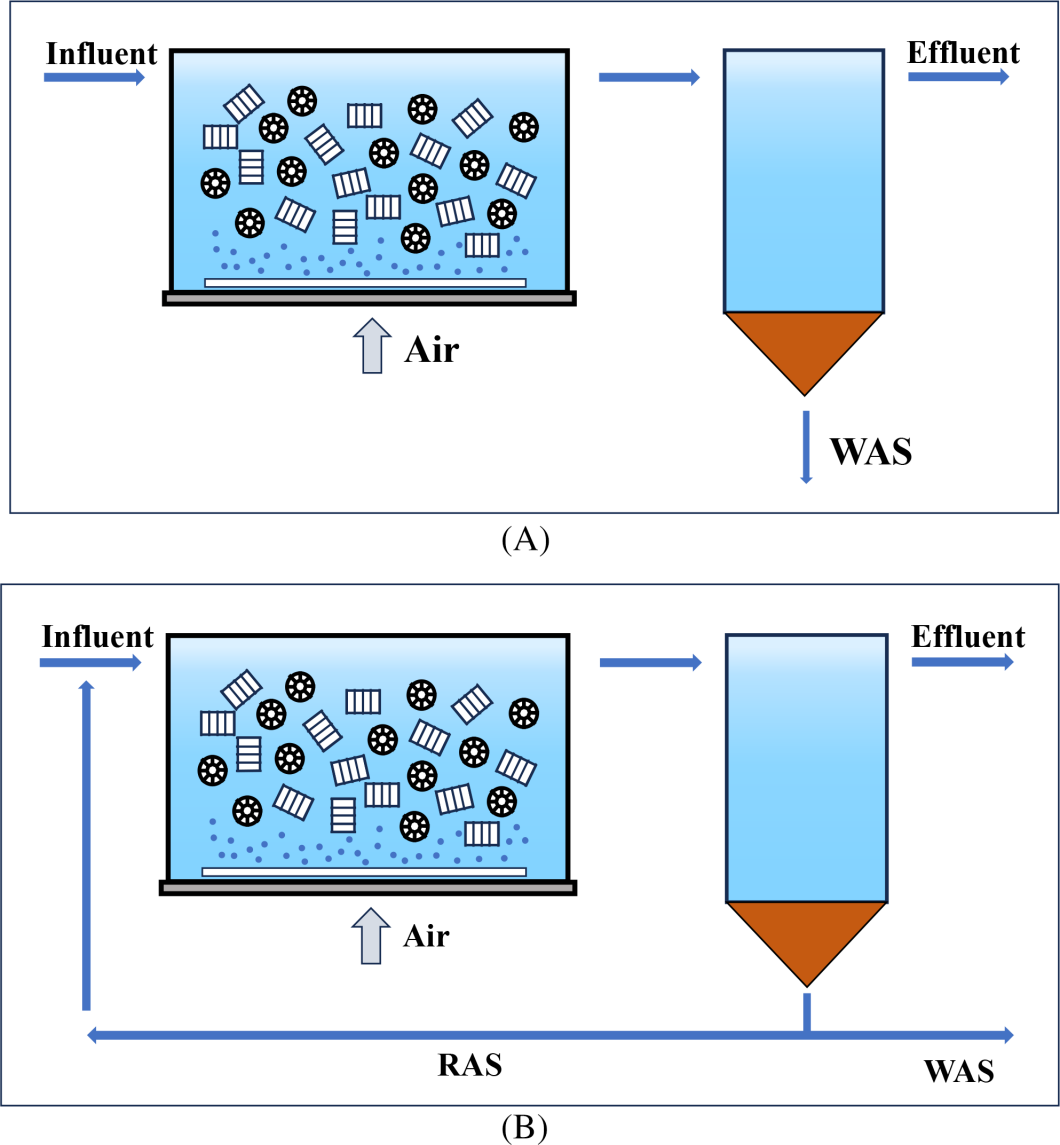
(A) MBBR followed by secondary settling tank (B) IFAS followed by secondary settling tank.

IFAS is often described as a hybrid system of suspended activated sludge and attached biofilm, where a high specific surface area carrier media is injected into the anoxic or aerobic zone of the system, and the carrier media can be either free‐suspended or immobile (Di Trapani et al., 2010; Lariyah et al., 2016). As shown in Figure 1, the main difference between IFAS (Figure 1B) and moving bed biofilm reactor (MBBR) (Figure 1A) is the presence or absence of return activated sludge, and Waqas et al. (2020) consider IFAS as an extension/upgrade of MBBR. In summary, the IFAS process is a system in which the attached and suspended biomes function synergistically, independent of the form of biofilm or suspended sludge present. The synergistic effect of biofilm and activated sludge enhanced the nutrient removal capacity of the system, especially for nitrogen fields (Gu et al., 2022; Vergine et al., 2018). IFAS reactors show more efficient nitrogen removal potential compared to moving bed biofilm reactors (Veuillet et al., 2014). The biofilm attached to the surface of the carrier media greatly increases the total biomass of the system and maximises the use of the reactor volume (Singh & Kazmi, 2016). It also enhances the impact resistance of the system (influent loads, low temperatures, and toxic substances) (Singh & Kazmi, 2016). The long sludge age of the biofilm helps to retain slow‐growing microorganisms such as AOB and AnAOB in the system from flushing (Gallardo‐Altamirano et al., 2021). The hybrid system somewhat alleviates the competition between different microorganisms, and microorganisms with higher oxygen affinity, such as NOB and AOB are more likely to be enriched in the suspended sludge. On the contrary, bacteria such as AnAOB and heterotrophic denitrifying bacteria (HDB) are more inclined to grow on biofilm fields (Liu et al., 2017; Yang, Zhang, et al., 2017). Suspended sludge can also be selectively discharged to inhibit NOB growth due to the difference in growth rates of AOB and NOB, which are both enriched in suspended sludge (Frison et al., 2018; Laureni et al., 2019). Sludge‐biofilm coexistence also enhances the collaboration between different microorganisms, for example, AnAOB can obtain NO_2_^−^–N through partial nitrification of AOB and partial denitrification of HDB in the suspended sludge, which opens up more possibilities for realising the process of Anammox. Various anaerobic ammonium oxidation nitrogen removal pathways based on IFAS technology have been extensively studied, such as partial nitrification anammox, simultaneous nitrification anammox denitrification, and partial denitrification anammox.

This paper reviews the anammox technologies such as partial nitrification anammox (PNA), simultaneous partial nitrification anammox and denitrification (SNAD), and partial denitrification anammox (PdNA) based on IFAS in recent years, and describes the factors limiting the development of related processes as well as some discoveries in recent years. Meanwhile, the influencing factors of dissolved oxygen, influent carbon source, sludge retention time (SRT), temperature, microbial community, and NOB inhibition methods on IFAS‐anammox were introduced for an outlook of the future to be made.

## IFAS WITH ANAMMOX

### Partial nitrification anammox

**FIGURE 2:**
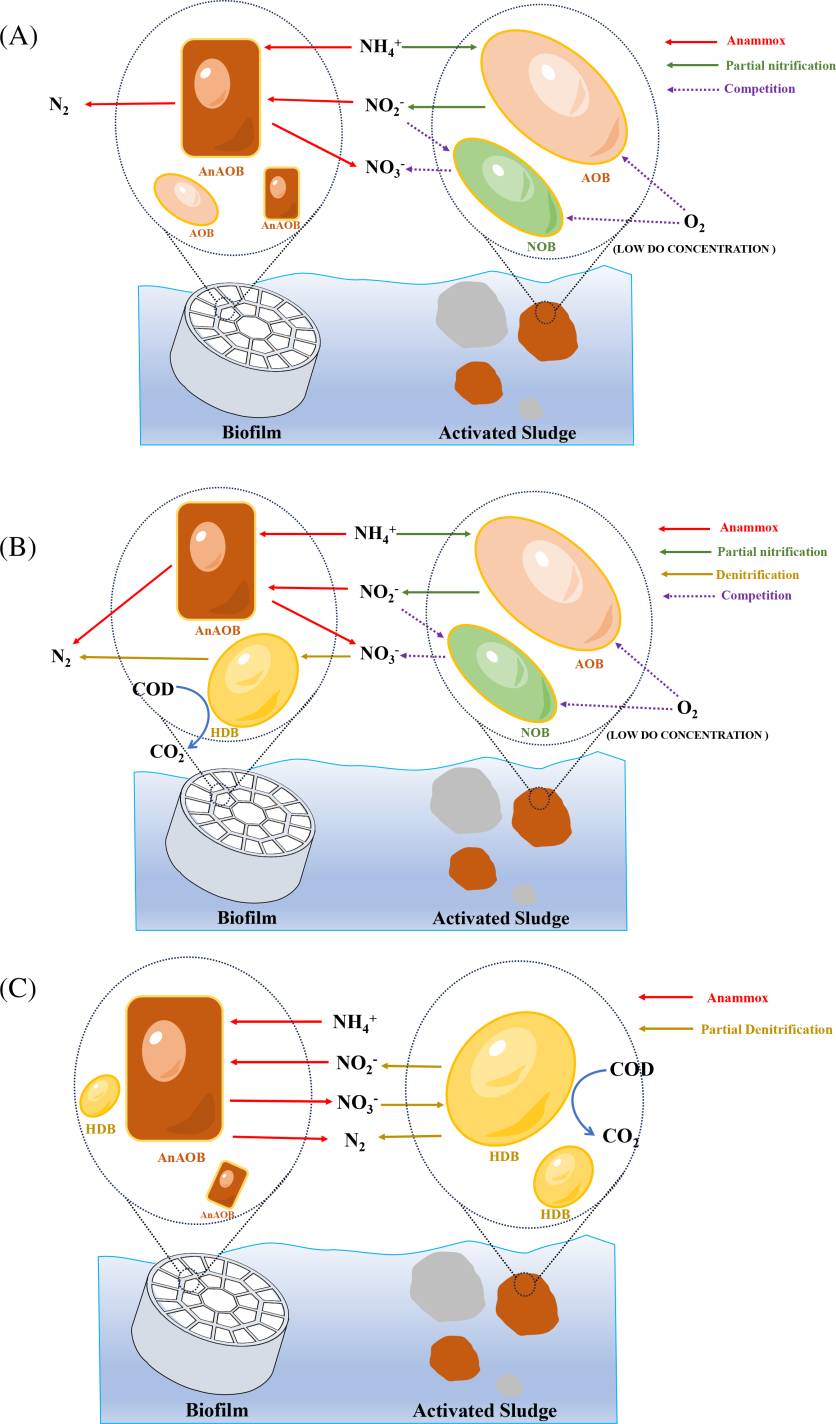
The substrate‐based interspecific competition and interspecific cooperation among functional microorganisms of different denitrification pathways in IFAS. (A) IFAS with PNA (B) IFAS with SNAD (C) IFAS with PdNA.

The PNA process is divided into two main steps (Figure 2A): AOB oxidises part of the NH_4_^+^–N to NO_2_^−^–N. Subsequently, NO_2_^−^–N and the remaining NH_4_^+^–N are converted to N_2_ by AnAOB (Strous et al., 1999). PNA, as a green and energy‐efficient autotrophic nitrogen removal technology, reduces the oxygen and carbon requirements of the process by about 60% and 100%, respectively, compared to conventional nitrification–denitrification (Zhang et al., 2017).

The synergistic effect of AOB and AnAOB and the inhibition of NOB activity are the key factors in achieving an efficient PNA process (Speth et al., 2016). The hybrid pattern of coexistence of suspended sludge and biofilm in the IFAS process helps to induce spatial differentiation of microbial populations and enhances the collaboration between AOB and AnAOB. Veuillet et al. (2014) found that AnAOB was mainly located in the biofilm (96% of total AnAOB) while AOB was mainly located within the suspended sludge (93% of total AOB) when comparing the ANITA™ Mox reactor in laboratory‐scale pure MBBR and IFAS mode. ANITA™ Mox in IFAS configuration is three to four times more efficient than the pure MBBR configuration due to the effective control of MLSS in the IFAS reactor and the spatial distribution of microorganisms in it, which facilitates access to substrates by AOB and AnAOB. AOB and NOB both grow in suspended sludge. They have different growth rates (μNOB < μAOB), which is a favourable condition for NOB elution. Laureni et al. (2019) found that NOB growth was further inhibited by operating a mixed PNA MBBR at 15°C, where the short SRT of the suspended sludge was scoured, allowing more nitrite to be used by the AnAOB in the biofilm, resulting in a ‘NO_2_^‐^‐sink’ phenomenon. A similar operating strategy with a nitrite source‐sink framework was found to help treat mainstream wastewater by Seuntjens et al. (2020). Therefore, the selective removal of suspended sludge from IFAS is an effective measure to enrich AnAOB and wash out NOB.

Recent studies have found that granular sludge formation in IFAS systems may be able to enhance NRE even further. Zhang, Liu, et al. (2015) used a pilot‐scale continuous flow IFAS reactor to treat high ammonium wastewater with an average denitrification efficiency of 80%. They observed the coexistence of flocculated sludge, granular sludge, and biofilm. The abundance of AnAOB in granular sludge (>100 lm) and biofilm was similar and four orders of magnitude higher than in flocculated sludge (<100 lm). Yang, Peng, et al. (2019) used an ANR process established by IFAS to treat high ammonium wastewater with a nitrogen removal rate (NRR) of 2.78 kgN/(m^3^ days). They found that the abundance of AnAOB was significantly higher in large flocs (*D* > 0.2 mm) than in small flocs (*D* < 0.2 mm) and biofilms during the stabilisation period, with as much as 51% of the AnAOB located in the flocs. In addition, the biofilm was similar to the microbial community of the large flocs, so the large flocs may have been formed by biofilm stripping. This is similar to the seeding‐effect previously reported by Di Trapani et al. (2010). Zhang, Li, et al. (2022) used intermittent aeration (aerobic 1 min/anoxic 5 min) and a low DO (0.20 mg/L) strategy in an IFAS system to make the reactor exhibit good denitrification performance with an average total nitrogen (TN) removal efficiency and TN removal rate of 80.6% and 0.13 kgN/(m^3^ days), respectively. Also, they found that the extracellular polymeric substance concentration of activated sludge increased greatly during the long‐term operation, which facilitated the formation of granular sludge. The maximum frequency diameter of the sludge flocs increased to 1000 μm. Both the AnAOB and the AOB were enriched in activated sludge and biofilm, and their abundances were 2.01% and 5.04% in the former and 2.43% and 2.45% in the latter, respectively. The granular sludge had better settling performance, easily maintained higher biomass concentrations, and was more stable compared to flocculated sludge. However, there are relatively few studies related to how granular sludge and biofilms cooperate in the PNA process to remove pollutants.

PNA has been widely used to treat high‐temperature, high‐concentration ammonium wastewater in wastewater treatment plant sidestream fields (Lackner et al., 2014). However, the long‐term stability of the PNA process remains a challenge under mainstream conditions with low ammonium concentrations (<100 mg N/L) and low temperatures (<25°C). IFAS as a hybrid system is highly preferred for operating PNA under mainstream conditions due to its more operability. The hybrid biofilm process developed by Hou et al. (2021) operating at a short hydraulic retention time (HRT) of 8 h successfully enriched AnAOB in the anoxic zone and effectively removed contaminants from the water. Malovanyy et al. (2015) treated mainstream wastewater by introducing suspended sludge into the MBBR reactor, and the denitrification efficiency increased from 36% ± 3% to 70% ± 4%. Trojanowicz et al. (2016) operated a similar system at 17°C, but the NRE was limited to 44%, mainly due to the nitrate produced by NOB activity at lower operating temperatures.

### Simultaneous partial nitrification anammox and denitrification

The PNA process, although exhibiting very high BNR removal efficiency, is inefficient in the simultaneous removal of carbon and nitrogen. The SNAD process has proven to be an effective single‐stage denitrification technology for the simultaneous removal of NH_4_^+^–N as well as organic matter from wastewater. The process (Figure 2B) starts with the oxidation of NH_4_^+^–N to nitrite by AOB under limited oxygen conditions (DO <3 mg/L), followed by anammox through AnAOB then denitrification by HDB in an anaerobic environment containing organic carbon as an electron donor (Mishra et al., 2022).

Compared to completely autotrophic nitrogen removal over nitrite, SNAD shows superiority in the treatment of municipal wastewater. The coexistence of HDB and AnAOB in an anaerobic environment protects AnAOB from inhibition by DO and high chemical oxygen demand (COD) (Wang et al., 2016). However, it has been shown by Zhang et al., (2019) that in SAND systems, the BNR efficiency decreases when the influent COD concentration increases, and the BNR efficiency reduces when the DO concentration decreases. High COD concentrations promote the accumulation of HDB, leading to the inhibition of AOB and AnAOB activity. Wang et al. (2018) used a SNAD‐IFAS reactor to treat mainstream wastewater, and the SNAD‐IFAS process achieved an average TN removal efficiency of 72% ± 2% and an average COD removal efficiency of 88% for different COD/N influents. The results showed that the optimum COD/N for treating mainstream wastewater was 1.2 ± 0.2, AnAOB and HDB were the main microorganisms in the biofilm, while high COD/N (≥2.0) resulted in the proliferation of NOB and heterotrophic bacteria in the suspended sludge. Meanwhile, network analysis showed that AnAOB showed significant symbiosis with some heterotrophic bacteria (*Limnobacter*, *Bryobacter*) and this symbiotic mechanism could protect AnAOB from the harsh environment (oxygen, organic matter). Xu et al. (2021) coupled an enhanced biological phosphorus removal (EBPR) process containing denitrifying polyphosphate‐accumulating organisms (DPAOs) with SNAD into anaerobic hydrolytic acidification‐simultaneous partial nitrification, anammox, and denitrification/enhanced biological phosphorus removal (ANHA‐SNAD/EBPR) process and operated it successfully. AnAOB in the SNAD/EBPR process was present in the biofilm, and AOB, DPAOs, and denitrifying bacteria (DNB) were mainly present in the suspended sludge. The process was successful because the ANHA reduced the COD concentration and most of the remaining volatile fatty acids had little inhibitory effect on the bacteria in the subsequent process.

When low DO concentrations are maintained, the production of NO_2_^−^–N in the wastewater is very low, which inhibits the activity of HDB and AnAOB. Therefore, it is important to optimise and monitor DO levels during SNAD (Xu et al., 2018). Zheng et al. (2019) investigated the efficacy of the SNAD process for treating mainstream wastewater under different intermittent aeration modes, controlling DO concentrations of 3.50, 1.45, and 0.70 mg/L under controlled intermittent aeration of 20, 60, and 180 min, and achieving 0.17, 0.29, and 0.30 kg N m^−3^ d^−1^ nitrogen removal rate. In a subsequent study, Zheng et al. (2021) found that the denitrification activity of the biofilm remained constant under different intermittent aeration operation strategies and was highly resistant to this change. The denitrification performance and microbial communities of two different hybrid system SNAD processes in intermittent aeration mode were investigated by Azari et al. (2021). The intermittent aeration mode was effective in controlling the DO range and achieved good nitrogen removal performance. The results also showed that the IFAS mode was better than the mixed mode of granular sludge and activated sludge.

### Partial denitrification anammox

The PdNA process is more economical and environmentally friendly than conventional nitrification–denitrification because PdNA reduces the consumption of carbon sources by 40% (Bahtiar et al., 2020). As mentioned earlier, the mainstream PNA process is faced with low temperatures and low concentrations of ammonium influent, and the need to suppress NOB activity. The PdNA process (Figure 2C) combines partial denitrification (reduction of NO_3_^−^–N to NO_2_^−^–N by denitrifying bacteria) with anammox (Izadi et al., 2023). Schoepflin et al. (2022) showed that PdNA‐MBBR start‐up does not require high concentrations of ammonium and nitrite and can be initiated rapidly without any inoculation of anammox biomass. This is because nitrite, a key substrate for anammox, is produced by denitrification. So the technology avoids the key limiting factor for PNA and SAND development—inhibition of NOB activity—and can reduce potential N_2_O emissions (Izadi et al., 2023). The combination of IFAS with the PdNA process gives the process more flexibility and stability. Zhang, Peng, et al. (2022) successfully removed nitrogen from pharmaceutical wastewater using a hybrid bioreactor. The key bacteria of PdNA in the hybrid system were highly tolerant and rapidly adapted to the pharmaceutical wastewater, achieving a relatively constant TN removal efficiency of 81.2%. Yu et al. (2023) inoculated the IFAS reactor with partial denitrifying flocs and anammox biofilms at 25 ± 1°C, while the MBBR reactor was inoculated with partial denitrifying biofilms and anammox biofilms. It was found that IFAS outperformed MBBR in terms of denitrification performance. *Thauera* and *OLB8* were the main DNB in the partial denitrifying biofilm in the MBBR and partial denitrifying floc in the IFAS respectively. The weaker denitrification performance of the MBBR was attributed to the tendency of *Thauera* to selectively immigrate towards the anammox biofilm, which reduced some of the partial denitrifying activity in the partial denitrifying biofilm. In contrast, AnAOB and DNB were present in the activated sludge and biofilm, respectively, in the IFAS reactor, and the activated sludge retained a higher fraction of partial denitrifying activity. The authors suggest that IFAS may be a potential superiority of the PdNA process in engineering applications.

Specific factors such as the type of carbon source can greatly influence the development of microbial communities (Wang, Chen, et al., 2021). Also, the type of carbon significantly affects the performance of denitrification and the accumulation of nitrite (Zhang, Zhang, & Chen, 2020). Ladipo‐Obasa et al. (2022) studied IFAS pre‐anoxic PdNA using fermentation broth as a carbon source to treat mainstream wastewater, which showed that high PdNA efficiencies (up to 38% PdNA efficiency and PdNA rates up to 1.2 ± 0.7 g TIN/m^2^ days) could be achieved even with low‐quality fermentation broth, compared to conventional nitrification and denitrification, and that the use of PdNA can save 48%–89% of methanol or other carbon sources when used with fermentation broth. Also, they confirmed that the amount of nitrate accumulation was the main factor driving PdNA efficiency.

## FACTORS AFFECTING THE DENITRIFICATION PROCESS OF IFAS

### Dissolved oxygen (DO)

Singh et al. showed (2016) that DO significantly affects IFAS reactor performance. The complete denitrification process involves a variety of microorganisms with very different personalities, which have different oxygen requirements. In the case of AnAOB, the optimal DO range is between 0.6 and 0.8 mg/L, while DNB requires a zero oxygen environment (Yin et al., 2016). The DO half‐saturation coefficients of AOB and NOB were in the range of 0.2–0.4 mg/L and 1.5–2.0 mg/L, respectively, which is important for NOB inhibition (Mishra et al., 2022). For specific processes, the PNA process requires DO concentrations ranging from 1.5 to 3.5 mg/L, while the SNAD process requires DO concentrations of 0.4 to 1.2 mg/L (Yang et al., 2015; Zou et al., 2018). You et al. (2020) suggested that DO should be controlled below 0.7 mg/L to accelerate the coupling process of PdNA.

As illustrated in Table 1, the intermittent aeration strategy is generally used to control the DO concentration in the PNA and SNAD processes. Because of the different DO requirements of AOB and NOB, the operating strategy of intermittent aeration and low DO can effectively inhibit NOB activity. Xu et al. (2020) found that DO concentration and duration of anoxia during aeration significantly affected the extent to which NOB was inhibited. Compared to high DO levels (1.5–1.8 mg/L), NOB was more suppressed in the mixed system at low DO levels (0.5 mg/L). Also, reducing the anoxic time from 40 min to 20 min and maintaining low DO during the intermittent aeration cycle still suppressed NOB activity, resulting in a 40% increase in denitrification. The DO concentration was key to the delayed recovery of NOB activity during intermittent aeration. The low DO strategy also enhanced biomass separation, which also facilitated NOB inhibition. Rong et al. (2022), Pedrouso et al. (2019) also confirmed that intermittent aeration was effective in suppressing the activity of NOB in the reactor. Mehrani et al. (2022) considered DO setpoint and on/off ratio as the most critical parameters for NOB suppression. Seuntjens et al. (2020) tested the effect of aerobic sludge age (AerSRT_floc_), different innovative aeration patterns, and nitrogen loading on mainstream IFAS‐PNA. Under optimal conditions suspended sludge becomes a source of nitrite, while biofilm becomes a nitrite sink and successfully suppresses NOB. Chi et al. (2021) proposed a novel high‐frequency micro‐aeration (HFMA) mode (aeration frequency 15 times/h, DO ≤0.5 mg/L) that enhanced the PNA performance of the IFAS‐SBR reactor, and the HFMA conditions enhanced the expression of AnAOB with NH_2_OH as an intermediate metabolite and the material exchange of functional microorganisms. Thus, the nitrite accumulation rate, denitrification efficiency, and AnAOB abundance of the reactor were improved (20.88%, 20.11%, and 5.92% increase, respectively).

**TABLE 1:** Summary of intermittent aeration strategy and performance.

IFAS progress	Type	Strategies (aeration/non‐aeration)	Aeration rate (L/h)	DO(mg/L)	Performance	References
Lab scale	P/NA	1 min/5 min	400	0.2 ± 0.1	The average TN removal efficiency and TN removing rate were 80.6% and 0.13 kg N/(m^3^·days), respectively	Zhang, Li, et al. (2022)
Pilot scale	P/NA	15 min/60 min	NA	1.5	The highest recorded capacity of AOB and AnAOB in biofilm was 1.4 gN/m^2^ days and 0.5 gN/m^2^ days, respectively, reaching 51% in nitrogen removal efficiency	Trojanowicz et al. (2016)
Lab scale	SNAD	1 min/10 min	120	0.4 ± 0.1	The TN removal efficiency achieved 72% ± 2% and COD removal efficiency was 88%	Wang et al. (2018)
Lab scale	SNAD	180 min/20 min	120	0.7	The average removal rate of total inorganic nitrogen (TIN) was 0.42 (kgN/m^3^/days). High DO and long aeration period may lead to a slight decrease in the abundance of denitrifying bacteria and anammox bacteria	Zheng et al. (2021)
Lab scale	SNAD	4 ± 1 min/21 ± 1 min	NA	1.2 ± 0.1	The highest NRE was 91% based on TIN. NRR achieved 0.01–0.15 kg N m^3^/days throughout the operation based on the TIN	Azari et al. (2021)
Lab scale	PNA	40 min/20 min	NA	0.5	NOB was more inhibited at low DO concentrations. Under optimised intermittent aeration, NRE reached 80%–89% with a NRR of 0.101 kg N/(m^3^ days)	Xu et al. (2020)
Pilot scale	PNA	15 min/45 min	1190	3.0	AnAOB was successfully maintained as the dominant flora, while the activity of NOB was inhibited and the effluent nitrate was kept low	Yang et al. (2015)
Pilot scale	PNA	4 min/1 min	NA	0.8	The abundance of NOB is only 0.1%–0.2% in this system.	Rong et al. (2022)
Lab scale	PNA	20 min/40 min	NA	0.58–0.64	Efficient nitrogen removal (72% ± 11%) was achieved at low temperature (21–15°C), with a NRR of 37 ± 3 gN/(m^3^ days) at 15°C. It effectively suppressed NOB activity, which accounted only for 10%–20% of the maximum potential activity	Pedrouso et al. (2019)
Mechanistic model	PNA	1 min/19 min	NA	0.2–0.25	Increased the NRE and NRR of total inorganic *N* (daily average) from 30% to >50% and 15–25 gN/m^3^ days, respectively	Mehrani et al. (2022)
Lab scale	PNA	1 min/1 min (15 times/h)	38.4	0.5	Improved NOB inhibition and anammox bacteria proliferation; increased nitrite accumulation rate, NRE, and abundance of anammox bacteria (16.34%, 18.71%, and 5.92%, respectively)	Chi et al. (2021)

### Influent carbon source

Organic carbon source or C/N significantly affects the growth and competition of microorganisms in the system, thus affecting denitrification efficacy (Bassin et al., 2016; Di Trapani et al., 2018; Zhang, Wei, & Wu, 2020). AnAOB is sensitive to influent organic matter, excessive C/N promotes the growth of heterotrophic bacteria and increases competition for space and oxygen between heterotrophic and autotrophic nitrifying bacteria (Bassin et al., 2012; Onnis‐Hayden et al., 2011). Some of the studies reported different levels of tolerance of anaerobic ammonium oxidation to C/N (Murthy et al., 2016; Ni et al., 2012). The reason for this may be due to the different degrees of tolerance to organic matter in different AnAOB. For example, *Candidatus Jettenia Asiatica*, *Candidatus Kuenenia Stuttgartensis*, *Candidatus Anammoxoglobus Propionicus*, and *Candidatus Brocadia Fulgidia* can utilise short‐chain fatty acids such as acetic acid and propionic acid (Al‐Hazmi et al., 2019; Dapena‐Mora et al., 2007; Huang et al., 2014). This suggests that the influence of the influent carbon source on the Anammox process depends on the microbial community and the characteristics of the influent organic carbon source.

For IFAS systems, the spatial separation of biofilm and activated sludge helps to increase the tolerance of autotrophic organisms to organic carbon and better enables the coupling of heterotrophic denitrification and anaerobic ammonium oxidation. Roots et al. (2020) found that increasing the C/N ratio from 2.3 to 3.1 resulted in an increase in nitrogen removal from 27% to 73%. This change was attributed to the transfer of nitrification from the biofilm to the suspended sludge. Shao et al. (2017) studied the IFAS‐SBR reactor performance and microbial community structure at different organic loads (COD of 500, 250, and 150 mg/L), with the reactors showing good ammonium and COD removal. The results showed that the utilisation of ammonium by microorganisms was closely related to the influent C/N ratio. Mannina et al. (2020) running IFAS‐UCT‐MBR found that low C/N ratios resulted in lower pollutant removal, while activated sludge was more active in removing organic carbon, and biofilm played a key role in nitrification.

It has been found (Wang, Gu, et al., 2022) that anaerobic ammonium oxidation dominates the coupled heterotrophic denitrification and anaerobic ammonium oxidation process when C/N < 3.75, and heterotrophic denitrification dominates when C/N > 6.5. Du et al. (2020) further reported the successful operation of the PdNA process in synthetic mainstream wastewater with C/N = 1.5 and improved denitrification efficacy. Yang, Guo, et al. (2019) studied the anaerobic IFAS process for the treatment of ammonium‐rich wastewater and found that efficient denitrification of more than 85% could be achieved for all C/N ratios in the range of 0.58–1.34. This is due to the spatial separation of microbial communities in the IFAS system and the presence of denitrifying activated sludge makes AnAOB resistant to high COD concentrations. Carbon to nitrogen rate is important for eliminating the competition between HDB and AnAOB for NO_2_^−^–N (Tang et al., 2014), and the limitation of the carbon source results in insufficient electron flow to nitrite reductase enzyme (Izadi et al., 2023). Setting the appropriate C/N allows HDB to express differences between feast (exogenous denitrification) and famine (endogenous denitrification) periods (Gong et al., 2013). Du et al. (2019) successfully mitigated the competition of HDB and AnAOB for nitrite and improved the efficiency of nitrogen removal using the step‐feeding organic carbon strategy.

Organic carbon source or C/N affects the functional microorganisms functioning in Anammox and determining the appropriate carbon source is important for coupling denitrification and anaerobic ammonium oxidation. IFAS as a hybrid system of biofilm and activated sludge seems to mitigate the effect of C/N to some extent.

### Sludge retention time

SRT is an important operating parameter for the anaerobic ammonium oxidation process because the growth rate of AOB in the suspended sludge is faster than that of NOB (μAOB > μNOB), which allows for the continuous removal of NOB through the disposal of waste sludge (Oleszkiewicz & Yuan, 2011). For example, the single reactor for activity Ammonia Removal Over Nitrite anaerobic ammonium oxidation (SHARON‐anammox) process for the treatment of sidestream anaerobic digestate SRT < 2 days. However, for mainstream wastewater, lower temperatures can lead to a decrease in μAOB, resulting in the AOB being discharged from the system under short SRT conditions (van Dongen et al., 2001). That is why the successful sidestream anaerobic ammonium oxidation process is difficult to apply to mainstream.

The biofilm in the IFAS process allows slow‐growing AOB to attach to its surface, because of the presence of suspended sludge, and can still inhibit NOB growth by operating with a short SRT (Mannina et al., 2018; Ødegaard et al., 2011). Di Trapani et al. (2013) found that the nitrification activity of biofilm was enhanced at low temperatures. The nitrification activity of suspended activated sludge in a mixed system was much higher than that of a pure suspended sludge system. Zhao et al. (2018) found that the nitrification of IFAS can occur in SRTs that are smaller than the nitrifying SRT. Both of these phenomena are due to the shear force generated by the external water flow and aeration, which leads to the shedding of biofilm with AOB in the surface layer (seeding‐effect) (Ødegaard et al., 2011). These properties of IFAS greatly reduce the loss of nitrosation due to short SRT. Laureni et al. (2019) showed that IFAS under low DO conditions allowed selective flushing of NOB over a wide range of SRT (6.8–24.5 days).

It is worth noting that DO concentration must be concerned when treating mainstream wastewater with IFAS‐Anammox for NOB inhibition via short SRT. Oxygen Penetration Depth is the maximum depth at which oxygen can penetrate the biofilm. Based on the DO content and the growth rate of NOB, the biofilm can be divided into three aerobic zones, which are the surface aerobic zone (0–*d*_1_), the intermediate aerobic zone (*d*_1_–*d*_2_), and the innermost aerobic zone (*d*_2_–*d*_3_). NOB can only colonise the intermediate aerobic zone. The narrower the intermediate aerobic zone, the more difficult it is for NOB to grow in the biofilm (Wang, Zheng, et al., 2022). This requires strict control of DO in the IFAS‐anammox system with short SRT to prevent NOB proliferation. Seuntjens et al. (2020) achieved an NRR of 122 ± 23 mg N L^−1^ d^−1^ and 73% ± 13% NRE by 7 days of aerobic SRT and two alternating DO setpoints (10 min at 0.07–0.13 mgO_2_L^−1^ and 5 min at 0.27–0.43 mgO_2_L^−1^).

### Temperature

Temperature variations have a significant effect on the activity of microorganisms involved in the BNR process. The appropriate temperature for denitrification of most AnAOB in wastewater treatment systems is between 30 and 40°C (You et al., 2020). It has been shown by Lotti et al. (2014) that the growth time of AnAOB increase**s** from 35 to 132 days when the temperature is decreased from 20 to 10°C. Therefore, sufficient biomass needs to be maintained to offset the adverse effects of temperature changes during denitrification. Zhou et al. (2016) investigated the effect of temperature decrease on the performance and microbial community of a laboratory‐scale hybrid Anaerobic oxic system at 25, 15, and 10°C, respectively. It was found that temperature variation had little effect on COD removal and slightly affected the removal of NH_4_^+^–N and TN. The results of high‐throughput sequencing analysis showed that the abundance and diversity index of microorganisms in suspended sludge reduces when the temperature is decreased from 25 to 10°C, while the abundance and diversity index of microorganisms in biofilm increases, and biofilm was more involved in nitrification reaction at low temperature. This may be a key factor for the stable removal of COD and TN at low temperatures. Rong et al. (2022) used the IFAS‐PNA process to treat the effluent from an anaerobic membrane bioreactor under seasonal temperatures (15–25°C). TN removal rates of 79.4%, 75.7%, and 65.9% were found at 25, 20, and 15°C, respectively. Low temperature inhibited both AOB and anaerobic ammonium‐oxidising bacteria (AnAOB), and the nitrogen removal was poor at 15°C because the activity of AOB decreased significantly. The successful retention of functional microorganisms and microbial isolation compensated for the adverse effects of low temperature, and NOB was well suppressed by intermittent aeration (4 min on and 1 min off) and low DO (~0.8 mg/L) strategies. The experimental results demonstrate the feasibility of using PNA as the mainstream denitrification process in temperate climates.

### Microbial community analysis

Table 2 summarises previous studies of the genus‐level abundance of functional microorganisms within biofilms and suspended sludge in IFAS systems. The stable and efficient performance of the system is highly dependent on the microbial community. In IFAS systems, the coexistence of suspended and attached biomass allows for the spatial separation of functional microorganisms. Numerous studies have shown (Martín‐Pascual et al., 2015; Ren et al., 2022; Zhang, Zhang, et al., 2015) that AnAOB and HDB tend to grow inside the biofilm and that AOB and NOB are more likely to be enriched on suspended sludge. Additionally, this results in a distinct contribution from biofilm and suspended sludge to nitrogen removal, increasing the system's effectiveness at removing nitrogen (Liu et al., 2017; Shao et al., 2018). Liu et al. (2018) found that the biofilm on the carrier media acts as a reserve capacity and when the substrate concentration exceeds the removal capacity of the suspended sludge, heterotrophic bacteria rapidly replace the autotrophic bacteria as the main active species in the biofilm, which contributes to the removal of decarbonization and ammonia.

**TABLE 2:** Summary of the genus‐level abundance of functional microorganisms in some IFAS systems.

Types	Operating conditions	Abundance of functional microorganisms in sludge	The abundance of functional microorganisms in biofilm	Influence factor	References
PNA	DO: < 0.3 mg/L *T*: 25°C HRT:6–12 h pH: 7.8–8.7	*Nitrosomonas*: 40.70%	*Nitrosomonas*: 5.66% *Candidatus Kuenenia*: 4.95%	Nitrogen loading rate	Liu et al. (2017)
PNA	DO:0.15–0.36 mg/L *T*: 22–25°C HRT: 7–10 h	*Nitrosomonas*: 7.7% *Candidatus Brocadia*: 0.9% *Comamonadaceae, Pseudomonas* and *Denitratisoma*: 5.2%	*Nitrosomonas*: 0.8% *Candidatus Brocadia*: 33% *Comamonadaceae*, *Pseudomonas* and *Denitratisoma*: 5.8%	Residual ammonium	Yang, Zhang, et al. (2017)
PNA	DO: 0.1–0.35 mg/L *T*: 27–29°C pH: 7.6–8.0 HRT: 7–9 h	*Candidatus Brocadia* in small flocs was 3.5%, *Candidatus Brocadia* and *Candidatus Kuenenia* in large flocs was 24.6%	*Candidatus Brocadia* and *Candidatus Kuenenia* in bioflim was 15.8%	HRT Nitrogen loading rate	Yang, Peng, et al. (2019)
PNA	DO: 0.2 mg/L Intermittent aeration *T*: 30°C HRT: 12 and 15 h	*Nitrosomonas*: 5.04% *Nitrosomonas*: 1.02% *Candidatus Kuenenia*: 1.87% *Candidatus Brocadia*: 0.14%	*Nitrosomonas*: 2.45% *Nitrosomonas*: 0.29% *Candidatus Kuenenia*: 2.38% *Candidatus Brocadia*: 0.05%	DO Intermittent aeration	Zhang, Li, et al. (2022)
SNAD	DO: 0.4 mg/L *T*: 25°C pH: 7.2 HRT: 0.75 days	When COD/N = 0.5–1.0 *Nitrosomonas*: 0.3%–0.9% *Nitrospira*: 1.0%–2.8% *Candidatus Kuenenia*: 0.5%–0.9% *Denitratisoma*: 0.1%–0.2% When COD/N = 1.0 *Blastocatella*: 34.8% *norank DS‐100*: 11.7% *norank SC‐1‐48*: 7.8% When COD/N = 2.0 *Nitrospira*: 34% *Hydrogenophaga*: 10.7% *Blastocatella*: 11.0% *Candidatus Kuenenia*: 0.06%	When COD/N = 0.5–1.0 *Candidatus Kuenenia*: 6.6%–10.5% *Candidatus Brocadia*: 0.6%–1.9% When COD/N = 1.0 *Candidatus Kuenennia* *Candidatus Brocadia* *Denitratisma* When COD/N = 2.0 *Nitrospira*: 7.5% *Candidatus Kuenennia* *Candidatus Brocadia* *Denitratisma*	COD/N	Wang et al. (2018)
SNAD	DO: 0.3–0.5 mg/L *T*: 27–30°C pH: 8 HRT: 48 h	*Nitrosomonas*: 4.5% *Denitratisma*: 1.1% *Candudatus Brocadia*: 0.6%	*Nitrosomonas*: 4.3% *Candidatus Brocadia*: 10.7% *Denitratisma*: 6.5%	DO Free ammonia Free nitrous acid	Xu et al. (2018)
SNAD	DO: 0.2–0.7 mg/L Intermittent aeration *T*: 30°C	In the flocs *Candidatus Brocadia*: 1.4% *Nitrosomonas*, *Nitrosolobus* and *Nitrosospira*: 3.65% *Nitrospira*: 0.6% In the granules *Candidatus Brocadia*: 11.9% *Nitrosomonas*, *Nitrosolobus* and *Nitrosospira*: 0.25% *Nitrospira*: 0.2%	*Candidatus Brocadia*: 6.6% *Nitrosomonas*, *Nitrosolobus* and *Nitrosospira*: 3.4% *Nitrospira*: 0%	DO Intermittent aeration Temperature	Azari et al. (2021)
PdNA	DO: 0.7–1.0 mg/L *T*: 17–29°C pH: 7–7.5 HRT: 48 h	*Thauera*: 57.7%% *Flavobacterium*: 20.8% *Candidatus Brocadia*: 0.1%	*Thauera*: 43.6% *Flavobacterium*: 6.8% *Candidatus Brocadia*:1.6%	Pharmaceutical wastewater	Schoepflin et al. (2022)
PdNA	DO: 0.7–1.0 mg/L *T*: 25°C pH: 7–8 HRT: 8 h	*OLB8*: 1.02%–20.54% *Pseudomonas*: 0.05%–6.34% *Thauera*: 5.05%–0.26%	*Candidatus Brocadia*:23.32% *SBR1031*: 6.93% *SC‐I‐84*: 4.15% *JG36‐GS‐52*: 2.29% *Candidatus Magasanik*: 2.49%	NA	Wang, Chen, et al. (2021)
SNAD‐PBR	DO: 0.4–1.2 mg/L *T*: 39°C pH: 7.8 HRT: 5 days	From 110 days to 210 days *Nitrosomonas*: 3.2%–1.7% *Nitrospira*: 0.5%–0.2% *Candidatus Brocadia*: 0.1%–1.5% *Denitratisoma*: 1.9%–0.9%	From 110 days to 210 days *Candidatus Kuenenia*: 0.1%–1.1%, *Nitrospira*: 6.5%–0.8% *Nitrosomonas*: 0.4%–0.3% *Denitratisoma*: 0.3%–1.9%	DO Reflux ratio COD/N	Zou et al. (2018)
PdNA	DO: 0.5 mg/L Intermittent aeration *T*: 20–26°C HRT: 6–8 h	*Candidatus Brocadia*: 0.1% *Nitrospira*: 0.6%	*Candidatus Brocadia*: 0.7% *Nitrospira*: 1.0%	DO Intermittent aeration	Xu et al. (2020)
PNA	DO: 0.8 mg/L *T*: 25, 20, and 15°C pH: 6.67–7.28 HRT: 4 h	*Nitrosomonas*: 16.4%, 3.4%, and 3.9% in temperature 25, 20, and 15°C, respectively. *Uncultured ƒ_Chitinophagaceae Niabella*: 5.2%, 5.9%, and 5.5% in temperature 25, 20, and 15°C, respectively	*Nitrosomonas*: 5.6%, 1.0%, and 1.0% in temperature 25, 20, and 15°C, respectively. *Uncultured f_Chitinophagaceae*: 4.9%, 6.2%, 4.9%, in temperature 25, 20, and 15°C, respectively. *Candidatus Kuenenia*: 6.5%, 4.2%, and 7.6% in temperature 25, 20, and 15°C, respectively. *Candidatus Brocadia*: 3.3%, 2.6%, and 2.8% in temperatures 25, 20, and 15°C, respectively.	Temperatures	Rong et al. (2022)
PNA	*T*: 30°C HRT: 2.5 days pH: 7.38–7.91	*Candidatus Brocadia*: 0.3% in phases II, and III, respectively. *PHOS‐HE51*: decreased from 18.2% in Phase II to 23.7% in Phase III	*Candidatus Brocadia*: 15.7%, 19.5%, and 18.9% in phases I, II, and III, respectively. *PHOS‐HE36*: decreased from 38.2% in phase I to16.6% in phase II	High organic loading Ammonia loading	Yang, Guo, et al. (2019)
PNA	DO: 0.5–0.8 mg/L *T*: 20–30°C pH: 7.2 HRT: 24 h	*Nitrospira*: decreased by 99.6%	*Candidatus Brocadia*: 1.5% in oxic biofilm,0.1% in anoxic biofilm *Candidatus Kuenenia*: 1.7% in oxic biofilm, 0.5% in anoxic biofilm	Temperatures	Ren et al. (2022)
SNAD	DO: 0.4–1.3 mg/L Intermittent aeration *T*: 30°C HRT: 18–30	*Candidatus Brocadia*: 2.93% *Uncultured_f_Nitrosomonadaceae*: 2.4%	*Candidatus Brocadia*: 7.67%	DO Intermittent aeration C/N	Du et al. (2021)

However, the slow growth rate of microorganisms such as AOB and AnAOB makes the rapid start‐up of the system a challenge. Young et al. (2017) used IFAS carrier media for a post‐carbon removal system to enable successful and rapid start‐up (18 days) of the nitrification system of the MBBR system at low temperatures (6–8°C). Du et al. (2021) rapidly established an IFAS‐SNAD system by inoculating nitridation‐suspended sludge in a small pilot‐scale reactor, with TN and COD removal rates of 92.8% and 78.8%, respectively. During the period, NO_3_^−^–N was generated excessively under continuous aeration conditions, but the NOB activity was suppressed and solved by intermittent aeration and a temporary increase in ammonium influent concentration. *Candidatus Brocadia* was identified as the dominant AnAOB with an abundance of 2.93% and 7.67% in suspended sludge and biofilm, respectively. Li et al. (2021) achieved a rapid start‐up (about 1 month) of P/NA‐IFAS by using a pre‐cultivated anammox biofilm. The rapid start‐up was attributed to the initial separation of AOB in the suspended sludge and AnAOB in the biofilm, which developed faster in the pre‐cultivated system and was nearly 10 times higher than in the bare‐media system without pre‐culture, and the abundance of AnAOB in the pre‐cultured system (2.2%) was much greater than in the bare‐media system (0.5%).

Recent studies have found that anoxic biofilms may be a suitable choice for the enrichment of AnAOB because it provide favourable conditions (low DO and low organic carbon concentration) (Cao et al., 2016; Ma, Qian, et al., 2017). According to the Xi'an wastewater treatment plant (Wang, Liu, et al., 2021) macrogenome report, AnAOB, and DNB were genetically abundant. The abundance of DNB in the anoxic tank was 5.9 times higher than that of AOB, and AnAOB was 8.9 times higher than that of the normal anaerobic‐anoxic‐aerobic (A^2^O) process. Li et al. (2019) improved nitrogen removal capacity in an anaerobic–anoxic–aerobic process wastewater treatment plant over 2 years of operation by applying moving carriers to the anoxic zone. The results showed that the improved nitrogen removal was associated with the enrichment of AnAOB on the media in the anoxic zone, and the abundance of AnAOB on the media was higher than that on the suspended sludge. The results of ^15^N‐stable isotope tracing tests indicated that anammox could be combined with denitrification through anoxic biofilms, which may have promoted the growth of AnAOB. Denitrification can be coupled with anammox through anoxic biofilms (PdNA process). Zhao, Chen, et al. (2021) combined a pilot‐scale A^2^O reactor with a biological aerated filter to treat low COD/N domestic wastewater. The results showed that the total inorganic nitrogen in the effluent was reduced from 17.1 to 9.8 mgN/L by the application of carrier media in the anaerobic and anoxic zones, which was associated with the enrichment of AnAOB and HDB on the biofilm in the anoxic zone, and the anoxic zone became the hot zone where the PdNA process occurred, contributing 34.1% of the TN removal. The application of carrier media in the anoxic zone may be a promising option for upgrading municipal wastewater treatment plants using the traditional suspended sludge process.

### Inhibition of NOB in mainstream wastewater

Successful anaerobic ammonium oxidation processes require sufficient substrates (NH_4_^+^–N and NO_2_^−^–N). The nitrogen in mainstream wastewater mainly exists in the form of ammonia nitrogen. So how to inhibit NOB and retain AOB has become a bottleneck limiting the development of Anammox (Zhang et al., 2019). Several effective methods have been used to inhibit NOB activity, such as low DO, intermittent aeration, SRT, organic matter competition (Choi & Jung, 2023; Yang et al., 2022), and the use of inhibitors such as free ammonia (FA) and free nitrite (FNA) (Wang et al., 2020; Wu, Gao, et al., 2022; Zhao, Zhao, et al., 2021). Suppression strategies such as SRT, low DO, and intermittent aeration have been discussed in detail above and are not repeated in this section. Some scholars have proposed to utilise the competitive relationship between aerobic heterotrophic bacteria and NOB to inhibit NOB. This is because NOB is more susceptible to inhibition by organic matter than AOB (Li, Li, et al., 2020). As the C/N ratio increased from 0 to 3 at 14–18°C, NOB activity gradually decreased by 66.5%, while AOB activity decreased by only 31.6%. Yang et al. (2022) rapidly established mainstream nitrosation (12 days) and stabilised nitrite accumulation at 92.1% in long‐term operation. The discovery may be able to bring new perspectives on NOB inhibition. FA/FNA concentrations are usually maintained in high ammonia concentration (500–1500 mg/L) wastewaters by adjusting pH so that it inhibits NOB (Vlaeminck et al., 2009). This is due to the different tolerance levels of AOB and NOB to FA/FNA (Kent et al., 2019). However, it is difficult to replicate for low ammonia concentration (20–50 mg/L) mainstream wastewater. Wang et al. (2014); (2017) achieved stable nitrite accumulation by returning part of the sludge to the reactor after sidestream FNA/FA treatment. Compared with sidestream FNA, sidestream FA treatment is more economical and applicable because sidestream FA only requires allowing a portion of the sludge to flow through the anaerobic digester without the need to construct a sidestream nitrification reactor (Wang et al., 2017). Xiong et al. (2023) found that the relative activity and abundance of NOB treated with sidestream FA decreased by 91.64% and 68.66%, respectively. They found through macrogenomics and macroproteomics that RNA polymerase, translation factor, and aa‐tRNA ligase were significantly down‐regulated. Ribosomal proteins and GTPases responsible for the binding of ribosomal proteins to rRNA or ribosomal subunits were also significantly down‐regulated. This suggested that protein synthesis and ribosome biogenesis were severely disrupted in NOB.

Despite the progress achieved for NOB inhibition, there are still many challenges to be faced. For low DO and intermittent aeration strategies, it has been found that a fraction of NOB (mainly Nitrospira) can adapt to long‐term low DO environments (Eng et al., 2018). Experimental results by Yuan et al. (2022) pointed out the difficulty of intermittent aeration in inhibiting the growth of Nitrospira, which led to the failure of part of the nitrification system. A low SRT will not only elute NOB but also AOB, which will reduce the efficiency of nitrogen removal. High C/N may increase the risk of heterotrophic outbreaks. Several reports indicate that NOB gradually adapts to FNA and FA inhibition (Duan, Ye, Lu, & Yuan, 2019; Ma, Yang, et al., 2017). Li, Duan, et al. (2020) showed that Nitrotoga is highly resistant to FA inhibition compared to Nitrospira and Nitrobacter. In addition, Duan, Ye, Wang, et al. (2019) found that NOB in the influent leads to failure of NOB suppression by sidestream FA/FNA treatment, and they concluded that pretreatment of the influent such as primary settling and high rate activated sludge is necessary.

In the long‐term treatment of mainstream wastewater, to ensure stable nitrite accumulation, due to the use of complex means to inhibit NOB. For example, sidestream FA is used to inhibit the activity of NOB, while the influent water is pretreated and supplemented with operational means (e.g., short SRT with low dissolved oxygen, intermittent aeration, etc.) based on the characteristics of the wastewater and microbial community to continuously and effectively inhibit NOB. The IFAS process is perhaps the best option for NOB inhibition using a composite approach. This is because it has more room to manoeuvre as a system of suspended sludge and biofilm co‐mingling. However, it requires researchers to understand more deeply the mechanism of NOB inhibition by different methods.

## CONCLUSION

The IFAS technology, as an extension of MBBR technology, combines suspended and attached biomass, and the separation of biomass provides more operational space for the retention of anaerobic AOB and the suppression of NOB. Therefore coupling of the IFAS process with anaerobic ammonium oxidation technology has a promising future. However, this technology still faces many dilemmas, such as NOB suppression, seasonally varying influent and temperature in mainstream wastewater, and slow start‐up.

In the future, many factors affecting IFAS and anaerobic ammonium oxidation, such as temperature, dissolved oxygen, C/N ratio, and so forth, should be investigated in more depth, and the related operational strategies should be optimised. The granular sludge formed in IFAS may be a beneficial tool for enhanced denitrification in the future, and subsequent studies should provide insight into the causes of granular sludge formation in IFAS and the cooperative relationship between granular sludge and biofilm. In terms of practical application, the formation of anoxic biofilm by the application of media in the anoxic zone may be an economical and effective measure for coupling anaerobic ammonium oxidation in conventional wastewater treatment plants to improve nitrogen removal capacity. IFAS may be the best option for NOB inhibition using a composite approach, but this requires researchers to have a deeper understanding of the mechanism of NOB inhibition by different approaches.

## AUTHOR CONTRIBUTIONS

**Guang Li:** Conceptualization (lead); funding acquisition (lead); methodology (lead); resources (lead); writing – original draft (equal); writing – review and editing (equal). **Yunyong Yu:** Investigation (lead); project administration (equal); validation (equal); visualization (lead); writing – original draft (equal); writing – review and editing (equal). **Xingyu Li:** Investigation (equal); project administration (lead); validation (equal). **Hongsheng Jia:** Data curation (equal); software (equal). **Xiaoning Ma:** Data curation (equal); software (equal). **Prince Atta Opoku:** Writing – review and editing (equal).

## CONFLICT OF INTEREST STATEMENT

The authors declare no conflict of interest.

## Data Availability

Data sharing not applicable to this article.
